# Nuclear Phospho-SOD1 Protects DNA from Oxidative Stress Damage in Amyotrophic Lateral Sclerosis

**DOI:** 10.3390/jcm8050729

**Published:** 2019-05-22

**Authors:** Matteo Bordoni, Orietta Pansarasa, Michela Dell’Orco, Valeria Crippa, Stella Gagliardi, Daisy Sproviero, Stefano Bernuzzi, Luca Diamanti, Mauro Ceroni, Gabriella Tedeschi, Angelo Poletti, Cristina Cereda

**Affiliations:** 1 Center of Genomic and Post-Genomic, IRCCS Mondino Foundation, Via Mondino 2, 27100 Pavia, Italy; matteo.bordoni@mondino.it (M.B.); orietta.pansarasa@mondino.it (O.P.); stella.gagliardi@mondino.it (S.G.); daisy.sproviero@mondino.it (D.S.); 2 Department of Neurosciences, University of New Mexico Health Science Center, 1 University of New Mexico, Albuquerque, NM 87131, USA; micheladellorco@salud.unm.edu; 3 Dipartimento di Scienze Farmacologiche e Biomolecolari (DiSFeB) and Centre of Excellence on Neurodegenerative Diseases, Università degli Studi di Milano, Via Balzaretti 9, 20133 Milano, Italy; valeria.crippa@unimi.it (V.C.); angelo.poletti@unimi.it (A.P.); 4 Department of Medicina Diagnostica e dei Servizi, Fondazione IRCCS Policlinico San Matteo, Viale Camillo Golgi 19, 27100 Pavia, Italy; s.bernuzzi@smatteo.pv.it; 5 Department of Brain and Behavioural Sciences, University of Pavia, Via Bassi 21, 27100 Pavia, Italy; luca.diamanti@mondino.it (L.D.); mauro.ceroni@mondino.it (M.C.); 6 Unit of General Neurology, IRCCS Mondino Foundation, Via Mondino 2, 27100 Pavia, Italy; 7 Department of Veterinary Medicine, University of Milan, Via Celoria 10, 20133 Milan, Italy; gabriella.tedeschi@unimi.it; 8 Centro InterUniversitario sulle Malattie Neurodegenerative, Università degli Studi di Firenze, Genova, Roma Tor Vergata and Milano, Viale Morgagni 50, 50134 Firenze, Italy

**Keywords:** oxidative stress, ALS, SOD1, DNA damage, peripheral blood mononuclear cells

## Abstract

We already demonstrated that in peripheral blood mononuclear cells (PBMCs) of sporadic amyotrophic lateral sclerosis (sALS) patients, superoxide dismutase 1 (SOD1) was present in an aggregated form in the cytoplasmic compartment. Here, we investigated the possible effect of soluble SOD1 decrease and its consequent aggregation. We found an increase in DNA damage in patients PBMCs characterized by a high level of aggregated SOD1, while we found no DNA damage in PBMCs with normal soluble SOD1. We found an activation of ataxia-telangiectasia-mutated (ATM)/Chk2 and ATM and Rad3-related (ATR)/Chk1 DNA damage response pathways, which lead to phosphorylation of SOD1. Moreover, data showed that phosphorylation allows SOD1 to shift from the cytoplasm to the nucleus, protecting DNA from oxidative damage. Such pathway was finally confirmed in our cellular model. Our data lead us to suppose that in a sub-group of patients this physiologic pathway is non-functional, leading to an accumulation of DNA damage that causes the death of particularly susceptible cells, like motor neurons. In conclusion, during oxidative stress SOD1 is phosphorylated by Chk2 leading to its translocation in the nuclear compartment, in which SOD1 protects DNA from oxidative damage. This pathway, inefficient in sALS patients, could represent an innovative therapeutic target.

## 1. Introduction

Amyotrophic lateral sclerosis (ALS) is a fatal neurodegenerative disease characterized by progressive loss of cortical and spinal motor neurons, resulting in muscle denervation and paralysis, which lead to death within 3–5 years after diagnosis, normally due to respiratory failure [1,2]. ALS is the most common degenerative disorder affecting motor neurons in adults, with an incidence that ranges from 2 to 5 cases per 100,000 individuals worldwide. More than 90% of cases are sporadic (sALS), while 5 to 10% of cases have a familial origin (fALS) [3]. Genetic mutations were found in a huge number of genes, but only few of them are epidemiologically relevant, including: SOD1, encoding for the copper/zinc superoxide dismutase 1, two RNA binding proteins (RBPs), trans-activation response element (TAR) DNA binding protein (TARDBP) and fused in sarcoma/translocated in liposarcoma (FUS/TLS, herein referred to as FUS), and C9ORF72, containing a pathological hexanucleotide repeat expansion [4,5].

The maintenance of genome stability and integrity is fundamental for cellular viability especially in neurons. Neurons are highly susceptible to DNA damage, both single-strand breaks (SSBs) and double-strand breaks (DSBs), because they are post-mitotic cells with a high metabolic rate, and they are also vulnerable to oxidative stress, which is one of the sources of DNA damage [6,7]. Furthermore, in post-mitotic neurons, because of their lack of self-renewal and replication, there are lesser choices to repair SSBs compared to proliferating cells. Thus, SSBs more probably could be transformed into more genotoxic DSBs [6]. To date, genome damage and their inadequate repair have been linked to degenerating neurons in ALS patients; however, the underlying mechanisms remain unknown [8].

Furthermore, some evidence suggests that DNA repair pathways and the DNA damage response (DDR) are impaired in numerous neurological disorders, including ALS [9]. Two protein kinases, ataxia-telangiectasia-mutated (ATM) and ATM and Rad3-related (ATR), have a key role in the DDR pathways that respond to genotoxic stress. While ATM is essential for the DSB response, ATR is important in the proliferating cells surviving, due to its role in the response to replication stress. Despite differences in substrate specificity both ATM and ATR phosphorylate hundreds of protein targets at Ser/Thr-Gln motifs, regulating DNA repair, replication, transcription, cell cycle checkpoint signaling, and cell fate pathways, such as apoptosis or senescence [10,11,12]. In neurodegenerative disease, persistent accumulation of DNA damage and defective repair mechanisms could lead to alteration of gene expression and neuroinflammation [13]. Recent breakthroughs, that permit to unravel these pathways and their role in neurodegeneration, could open to new therapeutic approaches and targets, involving all the factors that play a key role in DNA repair.

Recent evidence suggests that also non-neural cells, such as peripheral blood mononuclear cells (PBMCs), could participate in triggering moton neurons degeneration, thus could be used in studying neurodegeneration [14,15]. Moreover, using PBMCs of ALS patients, we reported a discrepancy between low SOD1 protein concentration and high SOD1 mRNA expression level [16,17]. In 2013, Cereda and co-authors demonstrated that the “missing” protein has two fates: Accumulates in cytoplasmic aggregates or relocates in the nuclear fraction, hence two sub-groups of sporadic patients were identified [15]. The presence of two sub-groups can explain the diverse severity of the phenotype of the pathology in patients. Unfortunately, the role of SOD1 impairment in DNA damage formation, i.e., DNA SSBs and DSBs has been controversially debated [18,19,20], and no data exist on its role in DDR.

Thus, in this study we aimed to investigate DNA damage in PBMCs of ALS patients, evaluating differences between the two sub-groups of patients, and in human neuroblastoma cells SH-SY5Y treated with hydrogen peroxide, used as a cellular model of ALS disease [17,21]. In addition, we wanted to evaluate the expression of DDR’s proteins, including ATM and ATR, and eventually to explore a new pathway in which they and SOD1 are involved.

## 2. Materials and Methods

### 2.1. Chemicals and Reagents

All chemicals were purchased from Sigma-Aldrich (Saint Louis, MO, USA) and were of analytical grade or the highest grade available. All reagents for cell culture were purchased from PAA Laboratories (Toronto, ON, Canada). Lipofectamine Plus was purchased from Life Technologies (Carlsbad, CA, USA). Rabbit anti-SOD1 (FL-154) (sc-11407); rabbit anti-Sp1 (pep2) (sc-59); mouse anti-pThr (H2) (sc-5367); mouse anti-Chk2 (A-12) (sc-5278); protein A/G plus agarose immunoprecipitation reagent (sc-2003) were purchased from Santa Cruz Biotechnology (Santa Cruz, CA, USA). Rabbit anti-alpha tubulin (GTX 112141) and rabbit anti-GAPDH (GTX 100118) were purchased from GeneTex (Irvine, CA, USA). Rabbit anti-pSer (ab9332) was purchased from Abcam (Cambridge, UK). Mouse anti-SOD1 (6F5) (MA5-15520) was purchased from Thermo Fisher Scientific (Waltham, MA, USA). Donkey anti-rabbit (NA9340) and anti-mouse (NA931) secondary peroxidase-conjugated antibody and Amersham ECL Select Western Blotting Detection reagent (RPN 2235) were purchased from GE Healthcare (Chicago, IL, USA). Anti-mouse CF™488A (sab4600036) and anti-rabbit CF™594 antibodies (sab4600099) were purchased from Sigma-Aldrich. Primers were purchased from Sigma-Aldrich.

### 2.2. Cell Culture and Treatment

Human neuroblastoma cells SH-SY5Y were maintained in Dulbecco’s modified Eagle/F12 medium (Life Technologies), supplemented with 15% fetal bovine serum (Life Technologies), 2 mM l-glutamine, 100 U/mL penicillin, 10 mg/mL streptomycin, at 37 °C in an atmosphere with 5% of CO_2_ and 95% humidity. Cells were monitored every day and, at the 80–90% of confluence, they were exposed to phosphate buffer saline solution (1× PBS) (T0) or treated with oxidative agents, such as 1 mM H_2_O_2_ (Sigma-Aldrich) for 30 (T30) and 60 (T60) min. SH-SY5Y were washed with cold 1× PBS, collected using a cell scraper, and centrifuged to allow the formation of a cellular pellet, ready to undergo the fractionated protein extraction. The interaction between Chk2 and SOD1 interaction was further confirmed by adding AZD7762, an inhibitor of both Chk1 and Chk2, to SH-SY5Y cells. Briefly, cells were exposed to 5 nM of AZD7762 and 1 mM H_2_O_2_ for 30 and 60 min. Successively, cells were washed, scraped and centrifuged as previously described.

### 2.3. Patients’ Enrolment

Patients affected by ALS were enrolled at the IRCCS Mondino Foundation in Pavia. Experiments were done using PBMCs isolated from 40 sporadic ALS (sALS) patients (mean age: 59.9 ± 9.4). ALS diagnosis was made according to the revised El Escorial Criteria [22]. SALS individuals harboring mutations in the SOD1, FUS/TLS, TARDBP, C9ORF72 and ANG genes were excluded from this study. Thirty-nine sex- and age-matched healthy volunteers, free from any pharmacological treatment (mean age: 54.7 ± 11), were recruited at the Transfusion Centre of the IRCCS Policlinico S. Matteo Foundation in Pavia. Healthy volunteers were all unrelated and the normal phenotype was confirmed by interviews based on personal health histories. The study design was examined by the IRBs of the enrolling Institutions. All individuals joined in the study signing the Consensus after reading Informative note. (see Section 2.14).

### 2.4. Isolation of PBMCs from ALS Patients and Healthy Controls

PBMCs were immediately isolated from peripheral venous blood by Histopaque^®^-1077 (Sigma-Aldrich) following the manufacturer’s instructions. Cells viability was assessed by trypan blue exclusion test. Aliquots of PBMCs were collected from each subject and processed for the following experiments.

### 2.5. Subcellular Fractionation

Both for PBMCs and SH-SY5Y, the subcellular fractionation was performed according to the method of Schreiber and colleagues [23], with some modifications. After cells have been washed with ice-cold 1× PBS, the cellular pellet was resuspended in ice-cold hypotonic lysis buffer (10 mM HEPES, pH 7.9, 10 mM KCl, 0.1 mM EDTA, 1 mM dithiothreitol, 0.5 mM phenylmethylsulfonyl fluoride, 1% of protease and phosphatase inhibitor cocktail). Cells were allowed to swell on ice for 25 min, after which 25 μL of 10% Nonidet NP-40 (Fluka, St. Gallen, Switzerland) was added. Samples were vortexed and centrifuged at the maximum speed. The supernatant, containing the cytoplasm proteins, was collected and stored at –80 °C until the use. The nuclear pellets were resuspended in ice-cold hypertonic nuclear extraction buffer (20 mM HEPES, pH 7.9, 0.4 M NaCl, 1 mM EDTA, 1 mM dithiothreitol, 1 mM phenylmethylsulfonyl fluoride, 1% of protease and phosphatase inhibitor cocktail), and incubated on ice for 20 min with agitation. The nuclear extracts were then centrifuged at the maximum speed for 5 min at 4 °C and the supernatant containing the nuclear proteins was collected and frozen at –80 °C.

### 2.6. Cell Transfection

To better distinguish cytoplasm and nuclear localization, we used chimeric fluorescent-tagged SOD1 proteins bearing either a nuclear export signal (NES) or a nuclear localization signal (NLS): YFP-NLS-wtSOD1 and YFP-NES-wtSOD1, respectively [18]. The plasmids YFP-NLS-wtSOD1, YFP-NES-wtSOD1 were used for transient transfections of SH-SY5Y cells using Lipofectamine 2000 (Life Technologies) following the manufacturer’s instructions, as also described in Crippa et al. [24]. About 1 × 10^5^ cells/mL were transfected using 0.7 μg of YFP-NLS-SOD1s or YFP-NES-SOD1s plasmids in addition to 2 µL of Lipofectamine 2000 (amount for 1 well of a 12 wells/multiwell).

### 2.7. Western Blotting Analysis

A Western blotting analysis was performed by SDS–polyacrylamide gel electrophoresis (SDS-PAGE). Thirty μg of nuclear and cytoplasm proteins were loaded onto 12.5% SDS–PAGE gel (Bio-Rad Laboratories, Hercules, CA, USA). After electrophoresis, samples were transferred to nitrocellulose membrane (Trans-blot, Bio-Rad Laboratories) using a liquid transfer apparatus (Bio-Rad Laboratories). Nitrocellulose membranes were treated with a blocking solution (5% of non-fat dry milk in TBS-T buffer, 10 mM Tris-HCl, 100 mM NaCl, 0.1% Tween, pH 7.5) to block unspecific protein binding sites and incubated with primary antibody overnight at 4 °C.

Immunoreactivity was detected using donkey anti-rabbit or anti-mouse secondary peroxidase-conjugated antibody (GE Healthcare; dilution 1:8000) and bands were visualized using enhanced chemiluminescence detection kit (ECL Select, Ge Healthcare). Both primary and secondary antibodies were removed from the membrane by means of stripping solution (mercaptoethanol, 2% SDS, and 62.5 mM Tris/HCl, pH 6.7), and then processed as described above. Densitometric analysis of the bands was performed using the ImageJ software (version number 1.51, http://rsb.info.nih.gov/ij/).

### 2.8. Immunocytochemistry

SH-SY5Y both at T0 and after treatment with 1 mM H_2_O_2_ (Sigma-Aldrich) for 30 (T30) and 60 (T60) min were harvested, washed with 1× PBS and suspended in RPMI-1640 medium (for PBMCs) and in DMEM-F12 medium (for SH-SY5Y). About 1 × 10^5^ cells were placed on a poly-L-Lysine slide (Thermo Fisher Scientific) and incubated at 37 °C to allow cell attachment to the slide. Cells were rinsed with 1× PBS and then fixed using a solution of 4% paraformaldehyde in 1× PBS [25]. Fixed cells were washed with 1× PBS and treated with a blocking solution (5% normal goat serum in 0.1% Tween-PBS) for 1 h to block unspecific protein binding sites, cells were then incubated ON at 4 °C with primary antibodies: Rabbit polyclonal anti-SOD1 (1:250 dilution), mouse monoclonal anti-SOD1 (1:250 dilution), mouse monoclonal anti-pThr (1:250 dilution) and rabbit polyclonal anti-pSer (1:250 dilution). Cells were washed with 1× PBS and incubated at room temperature for 1 h with secondary antibodies: CFTM 594 goat anti-mouse, (1:700 dilution) and CFTM 488A goat anti-rabbit (1:700 dilution) at room temperature for 1 h. Both primary and secondary antibodies were prepared in blocking buffer. Finally, samples were washed with 1× PBS, mounted with Prolong^®^ Gold antifade reagent with DAPI (Invitrogen, Carlsbad, CA, USA), dried, nail-polished and analyzed by confocal microscopy (Leica DM IRBE, Leica Microsystems Srl, Wetzlar, Germany).

### 2.9. Comet Assay

In order to study DNA damage, comet assay was performed in SH-SY5Y, non-transfected (NT) and transfected with NLS- and NES- SOD1, treated with 1 mM of H_2_O_2_ for 60 min, and in PBMCs of healthy controls and sALS.

Approximately 5 × 10^4^ cells, both SH-SY5Y and PBMCs, were collected and centrifuged at 4000 rpm for 5 min, the supernatant was discarded, and the pellet was suspended in 0.75% low-melting-point agarose (Sigma-Aldrich). The cell suspension was placed onto microscope slides coated with a layer of 1% agarose in 1× PBS. Slides with SH-SY5Y were immersed in cold lysing buffer (2.5 M NaCl, 0.1 M EDTA, 10 mM Tris, 1% DMSO and 1% Triton X-114, pH 12.5–13) for 30 min at 4 °C; while slides with PBMCs were immersed in alkaline lysis buffer (2.5 M NaCl, 0.1 M EDTA, 10 mM Tris, pH 10) for 1 h at 4 °C. Then, all slides were put in a horizontal electrophoresis tank, filled with cold electrophoresis buffer (0.3 M NaOH, 1 mM EDTA, pH 13), equilibrate for 40 min before starting electrophoresis (run conditions: 300 mA, 25 V, 30 min at 4 °C). Slides were neutralized with 0.4 M Tris, pH 7.5 for 15 min at 4 °C, covered and stored in a humidity chamber. After the addition of the nuclear dye Hoechst (Sigma-Aldrich), individual cells or ‘Comets’ were analyzed using a fluorescence microscope (Axio Imager 2, Zeiss, Oberkochen, Germany).

### 2.10. Immunoprecipitation

In order to evaluate the interaction between Chk2 and SOD1, we performed immunoprecipitation, that was performed according to Dell’Orco and co-authors [21] with minor modifications. After a pre-clearing phase, immunoprecipitation was carried out overnight at 4 °C using 2 μg of rabbit polyclonal primary antibody anti-SOD1 and anti-Chk2 for 300 μg of proteins diluted both in cytoplasm ice-cold lysis buffer (10 mM HEPES, pH 7.9, 10 mM KCl, 0.1 mM EDTA, 1 mM dithiothreitol, 0.5 mM phenylmethylsulfonyl fluoride, 1% of protease and phosphatase inhibitor cocktail) and in ice-cold nuclear extraction buffer (20 mM HEPES, pH 7.9, 0.4 M NaCl, 1 mM EDTA, 1 mM dithiothreitol, 1 mM phenylmethylsulfonyl fluoride, 1% of protease and phosphatase inhibitor cocktail) in the presence of 30 μL of protein A/G plus agarose (Santa Cruz Biotechnology). Samples were finally subjected to Western blotting for: Anti-Phosphoserine antibody (dilution 2 μg/mL in 5% bovine serum albumin (BSA)); anti-Phosphothreonine antibody (dilution 1:200 in 5% BSA); anti ChK2 (A-12) antibody (dilution 1:200 in 5% BSA). The negative control was obtained in the same conditions, in the presence of an irrelevant antibody with the same isotype of the specific immunoprecipitating antibody.

### 2.11. Mass Spectrometry

We analyzed the phosphorylation of SOD1 by means of mass spectrometry. To prepare mass spectrometry sample, 500 μg of both cytoplasm and nuclear proteins obtained from SH-SY5Y cells were immunoprecipitated by the Anti-FLAG M2 Magnetic Beads (Sigma-Aldrich) and eluted with the 3× FLAG peptide (Sigma-Aldrich). Samples were also treated with 50 U/µL of Phosphatase Alkaline (Sigma-Aldrich). Sample adequacy was verified by SDS-PAGE gel electrophoresis followed by silver staining of the gel and then processed for mass spectrometry. Upon immunoprecipitation, each sample was reduced with DTT, alkylated with 55 mM iodoacetamide at RT for 45 min and digested overnight with trypsin sequence grade, at 37 °C using a protease: protein ratio (1:20). The tryptic digest was desalted/concentrated on a ZipTipC18 (Millipore), and analyzed by mass spectrometry [26]. Nano LC-electrospray ionization-MS/MS analysis was performed on a Dionex UltiMate 3000 HPLC System with a PicoFrit ProteoPrep C18 column (200 mm, internal diameter of 75 m) (New Objective. Gradient: 1% ACN in 0.1% formic acid for 10 min, 1–4% ACN in 0.1% formic acid for 6 min, 4–30% ACN in 0.1% formic acid for 147 min, and 30–50% ACN in 0.1% formic for 3 min at a flow rate of 0.3 L/min. The eluate was electrosprayed into an LTQ-Orbitrap Velos (Thermo Fisher Scientific) through a Proxeon nanoelectrospray ion source (Thermo Fisher Scientific). Data acquisition was controlled by Xcalibur 2.0 and Tune 2.4 software (Thermo Fisher Scientific). The LTQ-Orbitrap was operated in positive mode in data-dependent acquisition mode to automatically alternate between a full scan (m/z 350–2000) in the LTQ-Orbitrap (at resolution, 60,000 (CID) or 30,000 (HCD); AGC target, 1,000,000) and subsequent CID and HCD MS/MS in the linear ion trap of the 20 (CID) or 10 (HCD) most intense peaks from full scan (normalized collision energy of 35%, 10-ms activation). Data Base searching was performed using the Sequest search engine contained in the Proteome Discoverer 1.3 software (Thermo Fisher Scientific). The following parameters were used: 10 ppm for MS and 0.5 Da for MS/MS tolerance, carbamidomethylation of Cys as fixed modification; phosphorylation of Ser, Tyr, and Thr, as variable modifications, trypsin (two misses) as protease. To generate the list of phosphosites reported in Table 1, we considered only the sites with the highest X Correlation value (Xcorr) in Sequest (1.5), the rank value of 1 and the best fragmentation pattern, selected manually after visual inspection of the MS/MS spectra. Three different tools for phosphorylation site prediction where applied: NetPhos 2.0 Server (DTU Health Tech, Lyngby, Denmark), Phosphosite plus (Cell Signaling Technology, Danvers, MA, USA) and Phosida (Max-Plank-Gesellschaft, München, Germany). Samples were analyzed by MS/MS before and after treatment with 50 U/µL of Phosphatase Alkaline to unambiguously verify the phosphosites listed in Table 1.

### 2.12. Real Time-qPCR

SH-SY5Y cell total RNA was extracted with the Trizol^®^ reagent (Invitrogen) using the manufacturer’s specifications and quantified by spectrophotometric analysis. One μg of RNA was reverse transcribed using an iScript cDNA synthesis kit (Bio-Rad Laboratories) according to the manufacturer’s recommendations. Primer and probe sequences: ATM Fw CAGGCGAAAAGAATCTGGGG, Rv GCACAAAGTAGGGTGGGAAAGC; ATR Fw TGAAAGGGCATTCCAAAGCG, Rv CAATAGATAACGGCAGTCCTGTCAC; CHEK1 Fw CAGGTCTTTCCTTATGGGATACCAG, Rv TGGGGTGCCAAGTAACTGACTATTC; CHEK2 Fw GCTATTGGTTCAGCAAGAGAGGC, Rv TCAGGCGTTTATTCCCCACC.

Real-time PCR was done on the LightCycler 480 II (Roche using iQ Sybr Green Supermix (Bio-Rad Laboratories). The qPCR incubations were run at 95 °C for 3 min, 95 °C for 15 s for 40 cycles, 60 °C for 45 s, 95 °C for 5 s, 65 °C for 1 min, then reach a peak of 97 °C and stay at 40 °C for 30 s. Averages of the Ct values from three replications for each sample with a standard deviation less than or the same as 0.15 was considered a valid result. Analysis of the ATM, ATR, CHEK1 and CHEK2 gene expression was made using GADPH (Fw CTTTTGCGTCGCCAG; Rv TTGATGGCAACAATATCCAC) to normalize. The amplification efficiencies for all genes were observed to be in the 90–110% range.

### 2.13. Statistical Analysis

All the experiments were performed at least three times. Values were expressed as means ± S.D. Statistical analysis was performed by One-Way Analysis of Variance (ANOVA) followed by Newman-Keuls Multiple Comparison as a post-hoc test (GraphPad Prism version 5, GraphPad Software, San Diego, CA, USA). Values were considered statistically significant when *p* values were <0.05.

### 2.14. Ethic Statement

All procedures performed in studies involving human participants were in accordance with the ethical standards of the institutional and/or national research committee and with the Helsinki declaration and its later amendments or comparable ethical standards. The study design was examined by the IRBs of the enrolling Institutions (Protocol n°375/04–version 07/01/2004).

## 3. Results

### 3.1. Cytoplasmic Aggregation of SOD1 Induce DNA Damage

**Figure 1:**
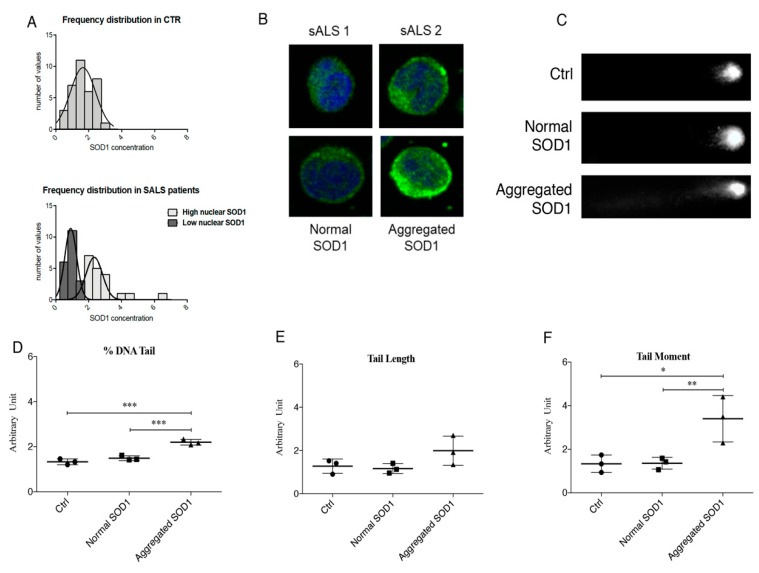
(**A**) Frequency Distribution of SOD1 in healthy controls (CTRL) (*n* = 40) and sporadic Amyotrophic Lateral Sclerosis (sALS) patients (*n* = 39). We found a bell-shaped distribution of the normalized values corresponding to SOD1 concentration for healthy controls, while a bimodal distribution was described for sALS patients confirming the presence of two sub-group. (**B**) Aggregation of cytoplasmic SOD1 in a subgroup of sALS patients observed by immunofluorescence. (**C**) Analysis of DNA damage in Peripheral Blood Mononuclear Cells (PBMCs) of control and patients with normal SOD1 and aggregated SOD1. Patients with normal SOD1 showed low damaged DNA (very weak Comet tail) similar to that observed in healthy CTRL; on the other hand, a very bright tail was observed in sALS patients characterized by cytoplasmic SOD1 aggregates. (**D**–**F**) Comet assay quantification by three different parameters: Tail length, % tail DNA and tail moment. Data were analyzed by ANOVA (*n* = 3), followed by Newman-Keuls Multiple Comparison Test; * *p* < 0.05; ** *p* < 0.01 and *** *p* < 0.001.

Considering our previous results, we first decided to expand our cohort of patients to confirm these data (Supplementary Figure S1A,B). A bell-shaped distribution of the normalized values corresponding to SOD1 was obtained for healthy controls, while a bimodal distribution was described for sALS patients confirming the presence of two sub-group. Furthermore, the aggregation of cytoplasmic SOD1 was identified by immunofluorescence only in a sub-group of sALS (Figure 1A,B). Once confirmed this tendency, we thus questioned about the possible effects of the reduction of normal soluble SOD1 and its aggregation in the cytoplasmic compartment.

To check DNA damage, we carried out the Comet assay on PBMCs of control and sALS patients characterized by high levels of cytoplasmic SOD1aggregates and by normal levels of soluble SOD1. In healthy control subjects (Figure 1A—CTRL) we found a total absence of Comet tails, in sALS1 patients with normal levels of soluble SOD1 we observed weak and very small Comet tails (Figure 1C—Normal SOD1). On the contrary, in sALS2 patients with high levels of cytoplasmic SOD1 aggregates we found intense staining in the region corresponding to the Comet tails (Figure 1C—Aggregated SOD1). Comet assay was then quantified by three different parameters: Percentage of tail DNA, tail length and tail moment (Figure 1D–F). The percentage of tail DNA significantly increase in patients with aggregated SOD1 (*p* < 0.001, Figure 1B). Moreover, we reported an increasing trend of the tail length in PBMCs of patients with high level of cytoplasmic SOD1 aggregates (Figure 1C). Finally, the tail moment, the most representative parameter, increased significantly in patients with cytoplasmic SOD1 aggregates respect to control and patients with normal SOD1 (*p* = 0.0145) (Figure 1D).

### 3.2. Activation of ATM/Chk2 and ATR/Chk1 Pathways in Treated SH-SY5Y

**Figure 2:**
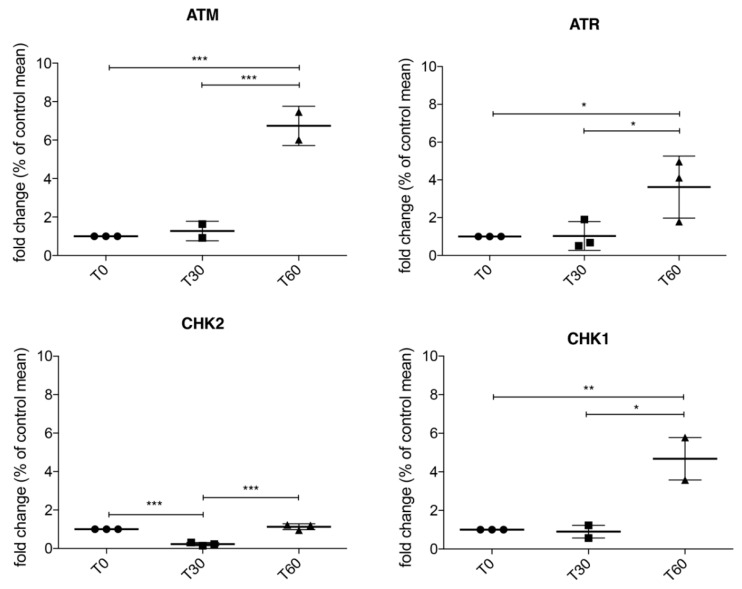
RT-qPCR in SH-SY5Y treated with 1 mM H_2_O_2_ for 30 and 60 min showed that ATM/Chk2 and ATR/Chk1 are actively transcribed after 60 min of oxidative stress, suggesting that SOD1 localization in the nuclear compartment is involved in DNA damage response. Data were analyzed by ANOVA (*n* = 3), followed by Newman-Keuls Multiple Comparison Test; * *p* < 0.05; ** *p* < 0.01 and *** *p* < 0.001.

In Supplementary Figure S1, we reported the aggregation of SOD1 in an in vitro model of oxidative stress-induced neuroblastoma SH-SY5Y cells treated with 1 mM H_2_O_2_ for 30 and 60 min. Confocal analysis revealed two different cellular populations: One with SOD1 nuclear distribution and one with the formation of cytoplasmic SOD1 aggregates (white arrows) which shows a lower concentration of nuclear SOD1. In response to oxidative stress induced DNA damage, various DNA repair and DNA damage response pathways are required to maintain genomic integrity [27,28]. ATM/Chk2 and ATR/Chk1 pathways are the two main DNA damage response systems induced by oxidative damaged DNA. To study the activation of this pathway, we used neuroblastoma SH-SY5Y cells. As reported in Figure 2, we observed an activation of both ATM and ATR serine/threonine kinase pathways at T60 (*p* < 0.001 and *p* = 0.0344), similarly we also observed a significant increase in mRNA levels of Chk1, while mRNA levels of Chk2 significantly reduces at T30 (*p* < 0.001), while a restore to normal level was reported at T60.

### 3.3. Chk2 Binds SOD1 in Presence of Oxidative Stress

**Figure 3:**
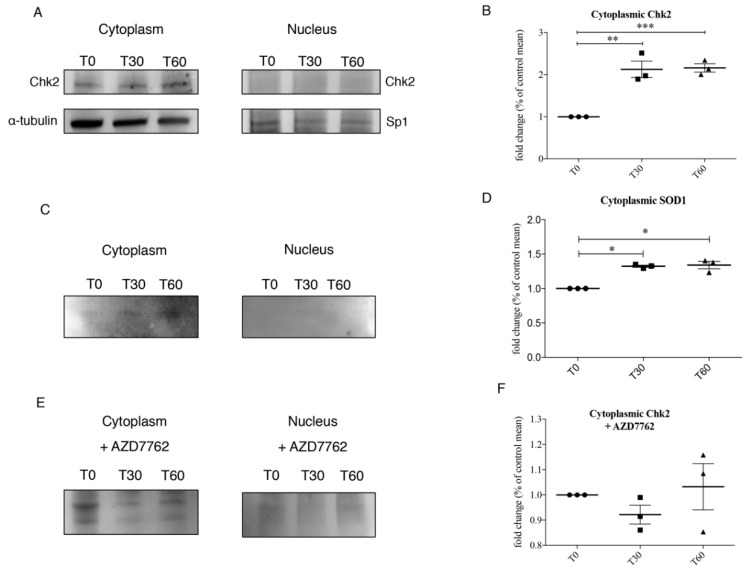
(**A**,**B**) Representative immunoblotting of cytoplasmic and nuclear Chk2 and quantification of Chk2 levels in the cytoplasmic compartment in SH-SY5Y at T0, T30 and T60. (**C**,**D**) Immunoprecipitation of Chk2 and quantification of cytoplasmic and nuclear SOD1 after oxidative stress. (**E**,**F**) The binding between Chk2 and SOD1 was further confirmed by immunoprecipitation also using AZD7762, a Chk1 and Chk2 inhibitor, to SH-SY5Y cells. After AZD7762 treatment no differences were observed in both nuclear and cytoplasm compartment. Data were analyzed by ANOVA (*n* = 3), followed by Newman-Keuls Multiple Comparison Test; * *p* < 0.05; ** *p* < 0.01 and *** *p* < 0.001.

We firstly confirmed in our cellular model the activation of Chk2 by western blot and we found (Figure 3A,B) a significant increase in Chk2 levels after 30 and 60 min of oxidative stress (*p* = 0.0010). Moreover, we performed Chk2 immunoprecipitation and we found a significant increase (*p* = 0.0327) in Chk2-SOD1 binding in the cytoplasmic compartment after 30 and 60 min of 1 mM H_2_O_2_ treatment (Figure 3C,D).

The interaction between Chk2 and SOD1 interaction was further confirmed by adding AZD7762, an inhibitor of both Chk1 and Chk2, to SH-SY5Y cells. Finally, AZD7762 treatment completely abolished SOD1-Chk2 binding in SH-SY5Y 1 mM H_2_O_2_ treated cells (T30 and T60) as showed in Figure 3E,F.

### 3.4. Oxidative Stress Stimulates SOD1 Phosphorylation

**Figure 4:**
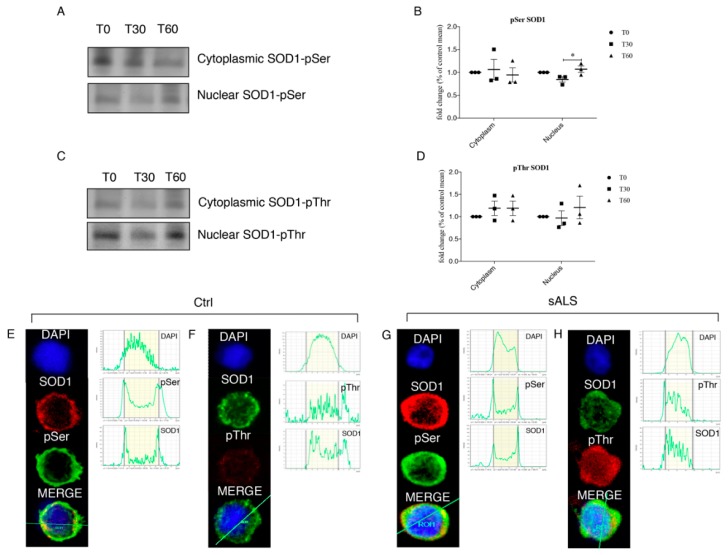
(**A**,**B**) 1 mM H_2_O_2_ treatment determines significant phosphorylation at Ser residue at T60 in the nuclear compartment. SOD1 was immunoprecipitated and representative immunoblotting for pSer was reported for the nucleus. (**C**,**D**) 1 mM H_2_O_2_ treatment induces significant phosphorylation also at Thr residue at T60 in the nuclear compartment. SOD1 was immunoprecipitated and representative immunoblotting for pThr was reported for the nucleus. Data were analyzed by ANOVA (*n* = 3), followed by Newman-Keuls Multiple Comparison Test; * *p* < 0.05. PBMCs from healthy controls and from a subgroup of sALS patients were analyzed for pThr, pSer and SOD1 by immunofluorescence followed by confocal microscopy analysis. (**E**,**F**) In healthy controls both pThr and pSer were not observed in the nuclear fraction; (**G**,**H**) in PBMCs of sALS patients we observed a bright signal of SOD1 and pThr in the nuclear compartment, while SOD1 and pSer co-localization showed a slight signal. Nuclei were visualized using the fluorescent nuclear dye DAPI (blue).

The activation of Chk2 and its interaction with SOD1 could account for the phosphorylation of the SOD1. We hence tested this hypothesis by SOD1 immunoprecipitation followed by the analysis of phosphorylation sites on cytoplasm and nuclear fractions from untreated (T0) and 1 mM H_2_O_2_ treated (T30 and T60) SH-SY5Y cells. As showed in Figure 4A,B, in the cytoplasm fraction, we found increased phosphorylation of SOD1 Ser residue. We observed a significant increased phosphorylation of Ser residues at T60 only in the nuclear compartment (*p* = 0.0236) and the same trend was observed for pThr even if it is not statistically significant (Figure 4C,D).

To verify if the phosphorylation observed in SH-SY5Y was also present in PBMCs of sALS, we carried out immunocytochemistry followed by confocal microscopy analysis. In healthy controls SOD1, pThr or pSer were not observed in the nuclear fraction (Figure 4E,F). Instead, in PBMCs of sALS patients we observed a slight co-localization between SOD1 and pSer (Figure 4G), and a much brighter signal for both SOD1 and pThr in the nuclear compartment confirming SH-SY5Y results (Figure 4H).

We tested a possible mechanism involving SOD1 nuclear re-localization by means of mass spectrometry. Mass spectrometry data revealed that, at cytoplasmic level, SOD1 is normally phosphorylated at several Ser and threonine Thr residues (Table 1) while a different pattern of SOD1 phosphorylation was observed in the nuclear compartment. In particular, phosphorylation at Thr residues presents differences in both number and type of aminoacid residue (Table 1).

### 3.5. Protective Role of SOD1 in Nucleus

**Figure 5:**
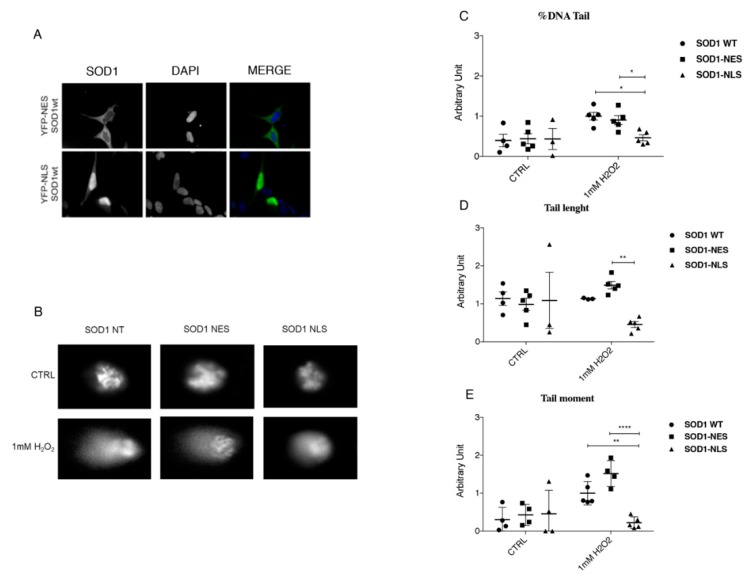
(**A**) Cytoplasmic and nuclear localization of SOD1; chimeric fluorescent-tagged proteins bearing either a nuclear export signal (NES; YFP-NES-wtSOD1) or a nuclear localization signal (NLS NLS; YFP-NLS-wtSOD1) in SH-SY5Y cells were used. (**B**) Protective role of nuclear SOD1 against DNA damage in SH-SY5Y cells; 60 min of 1 mM H_2_O_2_ Treatment induced marked DNA damage in both NT SOD1 and SOD1-NES in SH-SY5Y cells. In SOD1-NLS cells, instead, no comets were observed, indicating that minor or no DNA fragmentation occurred. (**C**–**E**) Comet assay quantification by means tail length, % tail DNA and tail moment were carried out. Data were analyzed by ANOVA (*n* = 3), followed by Newman-Keuls Multiple Comparison Test; * *p* < 0.05; ** *p* < 0.01 and **** *p* < 0.0001.

To demonstrate a new protective role of SOD1 in the nucleus, we performed Comet assay in SH-SY5Y cells using chimeric fluorescent-tagged SOD1 proteins bearing either a nuclear export signal (NES; YFP-NES-wtSOD1) or a nuclear localization signal (NLS ; YFP-NLS-wtSOD1) that allowed the translocation of SOD1 in the cytoplasmic and in the nuclear compartment (Figure 5A) [18].

Comet assay revealed marked DNA damage after 60 min of 1 mM H_2_O_2_ treatment in both SOD1-NT and SOD1-NES SH-SY5Y cells while in SOD1-NLS cells, instead, no comets were observed, indicating that no DNA fragmentation occurred (Figure 5B). To support our observation, in Figure 5 the three parameters of Comet assay, percentage of tail DNA (Figure 5B), tail length (Figure 5C) and tail moment (Figure 5D), were reported.

## 4. Discussion

Mutations in the gene encoding SOD1 were the first genetic cause of ALS to be identified [29] and are implicated in ~20% of all fALS and in ~1% of sALS cases. In our previous works we provided evidence for the involvement of wild-type SOD1 in sporadic cases, since a reduced SOD1 expression profile in lysates from PBMCs was observed [30]. On the other hand, abnormally high levels of SOD1 transcript were found in sporadic patients thus raising the question about the discordance between protein and mRNA expression levels [16]. To explain this discrepancy, we hypothesize a re-localization of the ‘‘missing’’ SOD1 protein in other cellular compartments, such as the insoluble fraction. Accurate information on the exact subcellular localization of a protein is critical for understanding its physiological and pathophysiological function, in particular aggregated SOD1 could be non-functional, generating oxidative stress that leads to DNA damage [30].

In PBMCs of sALS patients, we carried out a Comet assay and we clearly demonstrated the lack of DNA damage in the sub-group of sALS patients characterized by high levels of soluble nuclear SOD1, and an extensive DNA damage in sALS patients with high levels of insoluble/aggregate SOD1. This finding confirms our hypothesis that normal soluble SOD1 could have a potential protective role in DNA damage mediated by oxidative stress. On the contrary, aggregate SOD1 could be non-functional, leading to an increase of oxidative stress. Moreover, since SOD1 has a cytoplasmic peroxidase activity and it protects from oxidative stress, it could protect also DNA when it translocates to the nuclear compartment. Thus, we aimed to study such a pathway in our cellular model of neurotoxicity, SH-SY5Y treated with hydrogen peroxide.

We sought to confirm in our cellular model activation of DDR pathways after oxidative stress, and we found that the ATM/Chk2 and ATR/Chk1 pathways are both activated after 60 min of 1 mM H_2_O_2_ treatment. In particular, according to data reported in the literature [31] these two pathways are activated in a biphasic way, first ATM/Chk2 and then ATR/Chk1. Thus, we can postulate that, following H_2_O_2_-induced oxidative stress, different mechanisms crosstalk in order to coordinate DNA repair, cell cycle progression, transcription, apoptosis, and senescence.

Our observations lead us to suppose that the activation of DDR’s pathways could result in the phosphorylation of SOD1 thanks to their interaction. These data raised the hypothesis that oxidative stress lead to the activation of ATM/Chk2 and ATR/Chk1 pathways, in particular Chk2 could phosphorylate SOD1, as suggested by the work of Tsang and colleagues [32]. Mass spectrometry data directed our attention to SOD1 phosphorylation at several Thr and Ser residues. SOD1 phosphorylation could lead to the translocation of the protein in the nuclear compartment, in order to protect DNA from oxidative damage.

Thus, we finally evaluated the protection of SOD1 in the nuclear compartment by using specific localization tag. We confirmed that a higher concentration of SOD1 in the nucleus have a protective role in the DNA damage mediated by oxidative stress. On the contrary, when SOD1 is forced into the cytoplasm, it cannot protect DNA from damage.

These data should open a new scenario into the development of new therapies of ALS, in particular targeting molecules involved in oxidative stress protection. As yet to reported in cancer research [33], the nucleus could be a persuasive drug target, in particular by delivering nanoparticles directed in the proximity of the nucleus or against the nucleus itself. Targeted drugs that can reduce oxidative stress damage can have a pivotal role in delay the progression of the pathology.

## Figures and Tables

**Table 1:** List of phosphorylated peptides in cytoplasmic and nuclear compartment identified by MS/MS analysis. The Table reports only the sites with the highest X Correlation value (Xcorr) in Sequest (1.5), the rank value of 1 and the best fragmentation pattern, selected manually after visual inspection of the MS/MS spectra. Three different tools for phosphorylation site prediction where applied: NetPhos 2.0 Server, Phosphosite plus and Phosida. Samples were analyzed by MS/MS before and after treatment with phosphates to unambiguously verify the phosphosites listed in the Table.

Sample	PTMs	Peptide Sequence	Phosida	NetPhos 2.0	Phosphosite Plus	Ref. [26]
Cyt	S98	DGVADVSIEDSVISLSGDHCIIGRTLVVHEK	X	X	X	
	S102	DGVADVSIEDSVISLSGDHCIIGRTLVVHEK	X		X	
	S142	TGNAGSRLACGVIGIAQ	X			
Nuc	S34	PVKVWGSIKGLTE	X		X	
	T39	GSIKGLTEGLHGF				
	T58	DNTAGCTSAGPHF			X	X
	S59	NTAGCTSAGPHFN			X	X
	S69	PHFNPLSRKHGGP			X	
	S142	TGNAGSRLACGVIGIAQ	X			

## Supplementary Materials

The following are available online at https://www.mdpi.com/2077-0383/8/5/729/s1, Figure S1: Aggregation of SOD1 in SH-SY5Y cells treated with H_2_O_2_. Confocal analysis of SOD1 localization in SH-SY5Y treated cells.

## Abbreviations

ALS	Amyotrophic Lateral Sclerosis
ATM	Ataxia Telangiectasia Mutated
ATR	ATM and Rad3-related
Chk1	Checkpoint Kinase 1
Chk2	Checkpoint Kinase 2
DDR	DNA Damage Repair
DSB	Double Strand Break
fALS	familial Amyotrophic Lateral Sclerosis
FUS	Fused in Sarcoma
NES	Nuclear Export Signal
NLS	Nuclear Localization Signal
PBMC	Peripheral Blood Mononuclear Cell
PBS	Phosphate Buffered Saline
pSer	phosphoSerine
pThr	phosphoThreonine
RBP	RNA Binding Protein
sALS	sporadic Amyotrophic Lateral Sclerosis
Ser	Serine
SOD1	Superoxide dismutase 1
SSB	Single Strand Break
TARDBP	TAR DNA Binding Protein
Thr	Threonine
