# Supplementary material for: Nuclear Phospho-SOD1 Protects DNA from Oxidative Stress Damage in Amyotrophic Lateral Sclerosis
Source: J Clin Med. 2019 May 22;8(5):729. doi: 10.3390/jcm8050729 (PMC6572067; doi:10.3390/jcm8050729)
Supplement: Supplementary file 1 [file jcm-08-00729-s001.pdf]

Supplementary Figure

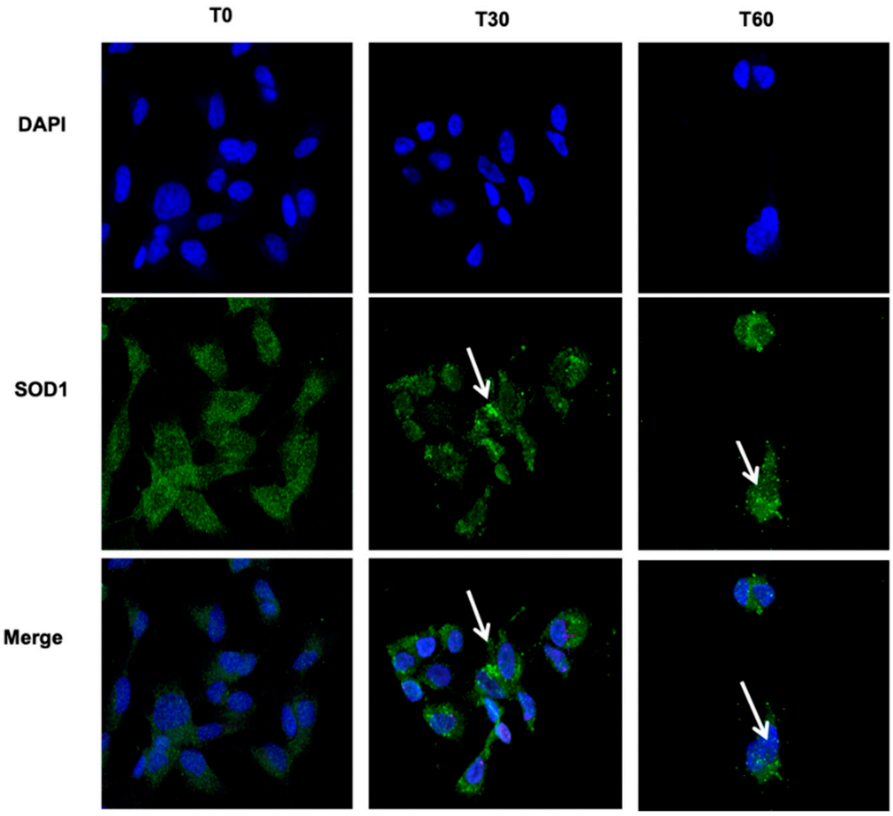

Figure S1. Aggregation of SOD1 in SH-SY5Y cells treated with H_2O_2. Confocal analysis of SOD1 localization in SH-SY5Y treated cells. After 30 and 60 min of treatment with H_2O_2 SOD1 shows a relocalization also in cell nucleus, especially after 60 min. It is evident the presence of two different populations: one with SOD1 nuclear distribution and one with the formation of SOD1 positive aggregates (withe arrows) which shows lower concentration of nuclear SOD1.
